# Generalizing the Wells–Riley Infection Probability: A Superstatistical Scheme for Indoor Infection Risk Estimation

**DOI:** 10.3390/e25060896

**Published:** 2023-06-02

**Authors:** Markos N. Xenakis

**Affiliations:** VTT Technical Research Centre of Finland Ltd., FI-02044 Espoo, Finland; mrksxenakis@gmail.com or markos.xenakis@vtt.fi

**Keywords:** indoor biosafety, infection risk estimation, COVID-19, *SEI* dynamics, Tsallis entropy, superstatistics, indoor-space epidemiology

## Abstract

Recent evidence supports that air is the main transmission pathway of the recently identified SARS-CoV-2 coronavirus that causes COVID-19 disease. Estimating the infection risk associated with an indoor space remains an open problem due to insufficient data concerning COVID-19 outbreaks, as well as, methodological challenges arising from cases where environmental (i.e., out-of-host) and immunological (i.e., within-host) heterogeneities cannot be neglected. This work addresses these issues by introducing a generalization of the elementary Wells-Riley infection probability model. To this end, we adopted a superstatistical approach where the exposure rate parameter is gamma-distributed across subvolumes of the indoor space. This enabled us to construct a susceptible (*S*)–exposed (*E*)–infected (*I*) dynamics model where the Tsallis entropic index *q* quantifies the degree of departure from a well-mixed (i.e., homogeneous) indoor-air-environment state. A cumulative-dose mechanism is employed to describe infection activation in relation to a host’s immunological profile. We corroborate that the six-foot rule cannot guarantee the biosafety of susceptible occupants, even for exposure times as short as 15 min. Overall, our work seeks to provide a minimal (in terms of the size of the parameter space) framework for more realistic indoor SEI dynamics explorations while highlighting their Tsallisian entropic origin and the crucial yet elusive role that the innate immune system can play in shaping them. This may be useful for scientists and decision makers interested in probing different indoor biosafety protocols more thoroughly and comprehensively, thus motivating the use of nonadditive entropies in the emerging field of indoor space epidemiology.

## 1. Introduction

As a consequence of the COVID-19 pandemic, humanity has faced an unprecedented crisis affecting every sphere of society, and causing a considerable health and economic burden. Unsurprisingly, several mitigation strategies have been proposed and implemented with the ultimate goal of controlling and, if possible, preventing the transmission of SARS-CoV-2 strains. Growing evidence suggests that air is the main pathway through which SARS-CoV-2 is transmitted [1,2,3,4,5,6,7,8]. Infection by SARS-CoV-2 is, thus, much more likely to occur indoors than outdoors. In particular, an infectious occupant can spread virus-containing aerosol particles (VCAPs) into the air via exhalation, resulting in the infection of susceptible occupants according to three main scenarios [9]: (a) the short-range airborne transmission scenario [10], where the spreader and the susceptible individual are in geometrical proximity; (b) the shared-room airborne scenario, where the spreader and susceptible individual are sharing the same indoor space, thus breathing from and exhaling into the same air container; (c) the longer-distance airborne transmission [11] scenario, where the spreader and the susceptible are not in geometrical proximity (i.e., they either do not share the same room or are far apart in an ample indoor space). Realization of any of these scenarios can trigger COVID-19 outbreaks of varying epidemiological magnitude depending on complexly interwoven biological (e.g., viral infectivity and the host’s immunological preparedness) and nonbiological (e.g., indoor space geometry and ventilation flow) factors. For example, there is a growing consensus that micrometer-sized VCAPs might underpin the transmission dynamics of so-called “superspreading events” [3,12,13], thus catalyzing the spread of SARS-CoV-2 in communities [8,14,15]. Although more than three years have passed since the beginning of the COVID-19 pandemic, the emergence of highly mutated and transmissible Omicron subvariants, such as XBB.1.5, poses significant threats to public health [16].

Mathematical modelling and simulation approaches to investigating airborne transmission indoors ultimately aim at developing comprehensive, quantitative methods for indoor infection-risk estimation (IIRE). The emergency of the COVID-19 pandemic accelerated multidisciplinary scientific efforts and provided a wealth of mathematical modelling approaches operating at various levels of abstraction, thus also claiming different degrees of biological plausibility. At a coarse-grained scale, SIR-like models can be insightful for understanding indoor infection dynamics at the occupant-group level (e.g., see [17,18,19,20]). On the other hand, refining the spatiotemporal scale leads to particle-based models attempting to shed light onto the transport mechanisms underlying airborne transmission and viral accumulation in the human body (e.g., see [21,22]). Typically, what all of these models have in common is an—at least—implicit connection between the indoor concentration (densities) of VCAPs and an infection rate parameter established via fluid mechanics. To this end, computational fluid dynamics (CFD) have been extensively applied to the study of airborne SARS-CoV-2 transmission pathways in different indoor environments (e.g., see [23,24,25,26]).

Due to the multiscale nature of the IIRE procedure, a crucial element of any modelling approach is the statistical frame upon which it relies to construct probability measures. Commonly, IIREs are obtained by either employing case-specific modifications of the classical Wells–Riley infection probability (WRIP) [27] or even developing entirely novel probabilistic approaches (e.g., see [28]). The WRIP’s primary assumption, inherited from Poissonian statistics, is that of a homogeneous (i.e., well-mixed) indoor air environment, implying that the transmission range is predominantly short, and exposure events are probabilistically independent of each other. Stated differently, the WRIP is based on the idea that, under steady-state conditions, indoor-air-environment-property gradients (in short, steady-state-invariant gradients), which are the underlying cause of observed statistical distances between a well-mixed and a not well-mixed, i.e., heterogeneous, VCAP spatial configuration, can be neglected. The realization, however, that omitting effects of steady-state-invariant gradients may return underrated IIREs in the vicinity of an infectious source [29] has led many researchers to reconsidering the applicability of the WRIP scheme by carefully localising it (e.g., see [25,26,28,30,31]). In practice, this approach can yield satisfactory IIREs at locations of high epidemiological interest, e.g., the breathing zone of susceptible individuals without, however, providing any systematic way to integrate local WRIPs into a nonlocal (i.e., macroscopic) measure for evaluating the biosafety of the indoor air environment as a whole. An additional layer of complexity that, to the best of our knowledge, remains unexplored, mainly due to insufficient knowledge of the innate immune system dynamics [32], can be added here by considering the possibility of heterogeneous within-host responses to inhaled VCAPs.

In this work, we present a superstatistical [33] solution to the problem of integrating local WRIPs into epidemiologically relevant macroscopic measures in terms of a gamma mixture model encoding spatial fluctuations of the exposure rate parameter in an arbitrary indoor space volume. The decisive step is to conceptualize a spatial-epidemiology model where susceptible occupants may receive VCAPs via distinct (i.e., noninteracting) pathways under the influence of indoor-air-environment stochasticities. Accordingly, we consider local exposure rates to reflect the joint yet probabilistically independent action of intrinsically stochastic airborne transmissions occurring simultaneously, but via different spatial routes. The core assumption accompanying this kind of spatial thinking is that fast- and slow-dynamics timescales co-exist and are well-separated: in a steady-state indoor air environment, fast dynamics is given by the emission and inhalation of VCAPs, while slow dynamics describes how an indoor-air-environment-property-gradient field may change. This implies that macroscopic changes in the VCAP (spatial) distribution occurring during a predetermined exposure period are forbidden as long as the steady-state assumption is satisfied. Our modelling procedures suggest a frameshift from Poissonian to Paretian statistics, thus leading directly to a *q*-exponential WRIP, with *q* denoting the Tsallis entropic index [34] and admitting the interpretation of the degree of heterogeneity associated with a given steady-state indoor air environment. From a spatial-epidemiology viewpoint, q>1 signals a nonvanishing transmission range, thus enabling construction of a distance-sensitive susceptible (*S*)–exposed (*E*)–infected (*I*) dynamical model where the magnitude of the rate at which occupants are transferred from the *E* subgroup to the *I* subgroup depends on immunological traits. Specifically, the Richards growth model [35] is employed to describe the dynamical relationship among viral accumulation, infection activation, and a hypothetical innate-immune-system defence mechanism orchestrated by neutralising antibodies (NAbs), and potentially enhanced by the interferon (IFN) system (for reviews covering the crucial roles that NAbs and the IFN system play in disrupting SARS-CoV-2 pathogenesis, see [36,37], respectively). This allows for investigating the interplay between out-of-host and within-host heterogeneities in a single model that, as we show, provides a simple tool for evaluating distance-based mitigation strategies such as the six-foot rule.

## 2. Model Construction

### 2.1. Preliminaries

We consider an enclosed space of rectangular volume *V* (m${}^{3}$) occupied by *N* randomly mixed individuals split into a group of susceptible individuals of size *S* and infectious individuals of size *F*. The indoor air environment was assumed to relax into a steady state. Infectious occupants act as virus spreaders by emitting VCAPs into *V*. Susceptible occupants can be exposed to the virus via airborne transmission, i.e., by inhaling air samples from $v_{br}<V$ containing VCAPs, with $v_{br}$ denoting the breathing zone volume, i.e., the air volume surrounding a susceptible occupant and determining their epidemiological status. The dynamics of VCAP emission and inhalation are paced by $\tau_{rel}$, denoting the fast-dynamics timescale. The breathing-cycle period gives the magnitude of $\tau_{rel}$, which is ≈3 (s). $\tau_{rel}$ also determines the time it takes for the local equilibrium density of the indoor air particles (thus also of VCAPs) to be restored. Accordingly, perturbations in the local density of VCAPs are expected to be damped out very quickly. The time over which macroscopic environmental changes can occur, i.e., the slow-dynamics timescale, is denoted with T. The magnitude of T determines the period over which an indoor-air-environment-property-gradient field might change. It is required that $T/\tau_{rel}\gg 1$. The epidemiological status of susceptible occupants may be probed at any time $t^{\prime }\in \left[t_{0},t^{\prime \prime }\right]$, where $\tau t^{\prime \prime }-t_{0}$ (h) is the occupancy time, i.e., the total time that *N* occupants spend in *V*. It is required that τ<T so that the steady-state assumption is satisfied at any $t^{\prime }$. The average maximum time that the breathing zone of a susceptible occupant is contaminated with at least one VCAP gives the exposure time; let this be denoted with $\tau^{\prime }\leq \tau$. The average maximum distance over which a VCAP can be transported during $\tau_{rel}$ delimits the transmission range; let this be denoted with ξ (m). ξ plays a similar role throughout this work to the one that the correlation length plays in superstatistical applications (e.g., see [38]). For simplicity, it is assumed that each VCAP contains an equal number of virions. An explicit connection with epidemiology is obtained via the notion of infectious quantum (IQ) (plural form abbreviation: IQa) [27] defined here as the critical number of VCAPs, $\Phi_{crit}\;\left(\mathrm{VCAPs}\right)\;\equiv 1\;\left(\mathrm{IQ}\right)$, that, once deposited in the body, is expected to activate an infection.

Table 1 aims to ease the reader by presenting key parameters and highlighting their interdependencies.

### 2.2. Airborne Exposure Risk Statistics

#### 2.2.1. Homogeneous Indoor Air Environment

If the indoor air environment is homogeneous (for a geometric description of a homogeneous indoor air environment, see Appendix A.1, a series of exposure events realised anywhere in *V* can be thought of as a Poisson process, where the probability that any pair of subsequent exposure events are timely separated by $t=t^{\prime }-t_{0}$ is given by the exponential probability density function (PDF):

(1) $$\begin{array}{c} p(t|\lambda )=\lambda \exp(-\lambda t), \end{array}$$

where λ (1/h) is a macroscopic rate designating the speed at which individuals are exposed to the virus via airborne transmission. It is common practice to consider substitution $\lambda =r\frac{Fw}{W\Phi_{crit}}$, where w>0 (VCAPs/h) is the rate at which VCAPs are emitted into *V* by an infectious occupant (i.e., VCAP exhalation rate), r>0 (m${}^{3}$/h) is the rate at which a susceptible occupant breathes air from *V* (i.e., volumetric inhalation rate), and W>0 (m${}^{3}$/h) is the rate at which clean air is supplied to *V* by a ventilation system [27]. The total number of VCAPs exhaled and inhaled during *t* by infectious and susceptible occupants is then given by Fwt and λt, respectively, and the steady-state VCAP density reads [27]

(2) $$\begin{array}{c} \rho =\frac{K}{V}=\frac{Fw}{W} \end{array}$$

from which it follows that

(3) $$\begin{array}{c} \lambda =r\frac{\rho }{\Phi_{crit}}. \end{array}$$

The cumulative distribution function (CDF) associated with Equation (1) gives the classical WRIP [27]:

(4) $$\begin{array}{c} P(t|\lambda )=1-\exp(-\lambda t), \end{array}$$

which is assumed to serve as a good approximation for the ratio $\frac{E}{S}$ [27], i.e., $P\left(t|\lambda \right)\approx \frac{E}{S}$, where *E* represents the number of susceptible occupants exposed to the virus after spending *t* time in *V* (in short, exposed occupants). For t=1 and λ=1, (4) returns an exposure risk of ≈63.2%, namely, ≈63.2% of susceptible occupants have been exposed to the virus.

#### 2.2.2. Heterogeneous Indoor Air Environment

**Figure 1:**
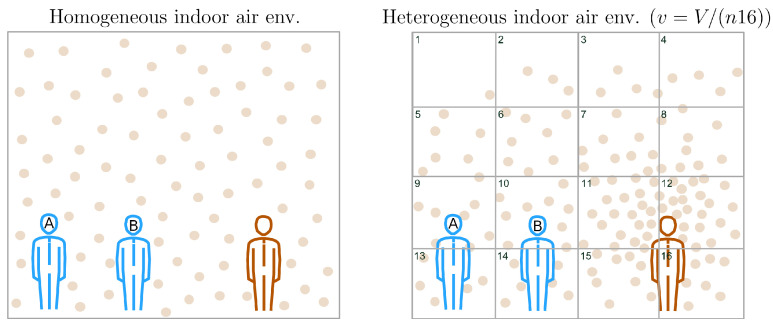
Schematic illustration of the proposed spatial-epidemiology model. We consider an indoor space of volume *V* occupied by one infector (i.e., F=1) and two susceptible (i.e., $S_{0}=2$) individuals, shown in red and blue, respectively. Round dots represent steady-state VCAP densities in *V*. The classical WRIP scheme assumes that the indoor air environment is homogeneous, i.e., that VCAPs are roughly uniformly spaced in *V* (see subfigure on the **left**). On the other hand, the generalized WRIP scheme does not rely on the homogeneity assumption (e.g., as we can see in the subfigure on the **right**, VCAP density can be higher near an infectious source). To systematically capture deviations from homogeneity, we partition *V* into i=1,2,…,Ω=n16 subvolumes of size v=V/Ω, where *n* denotes the number of subvolume layers used to fill *V*. For clarity, we illustrate only the front layer containing the first 16 subvolumes (subvolume boundaries are highlighted in black). Supposing that the epidemiological status of susceptible occupants is determined by a single surrounding subvolume (i.e., if κ=1), then the subvolumes indexed with i=9 and i=10 correspond to the breathing zones of susceptible occupants *A* and *B* with $\lambda_{9}=x_{9}$ and $\lambda_{10}=x_{10}$, respectively, representing the values of the corresponding realizations of λ with λ∼Gamma(1,μ)=Exp(μ) (see Equations (11) and (14)).

If the indoor air environment is heterogeneous (for a geometric description of a heterogeneous indoor air environment, see Appendix A.1, getting a similar closed-form expression for the WRIP to (4) requires updating our knowledge concerning the location of VCAPs in *V*. The first step in this direction is to refine the spatial resolution of the IIRE procedure on the basis of our knowledge concerning the value of ξ (to gain some insight on what the order of magnitude of ξ might be, see Appendix A.1, relationship (A2)). For this, let us assume that *V* can be partitioned into $\Omega \in Z_{+}$ nonoverlapping cubic subvolumes of size

(5) $$\begin{array}{c} v=\frac{V}{\Omega }\;\;\;\;\;\left(m^{3}\right), \end{array}$$

where Ω is chosen, so that $\sqrt[{3}]{v}\propto \xi$ (for an illustration, see Figure 1). With Equation (5) at hand, we can express *K* as $K=\sum_{i}^{\Omega }k_{i}$, where $k_{i}$ gives the number of VCAPs suspended in the *i*-th subvolume. By doing so, we have silently introduced a random variable, namely, the random variable *k* accounting for fluctuations of the local VCAP number, i.e., of the number of VCAPs suspended in a *v*-sized subvolume. Accordingly, $k_{i}$ denotes the *i*-th realisation of *k*. No assumption concerning the PDF of *k* is made except by requiring that the mean value of *k* be given by

(6) $$\begin{array}{c} \langle k\rangle =\frac{1}{\Omega }\sum_{i}k_{i}=\frac{K}{\Omega }, \end{array}$$

where angular brackets 〈·〉 indicate that the mean value calculation was performed over Ω subvolumes. *k* can only weakly fluctuate over $\tau_{rel}$; specifically, large-amplitude fluctuations of *k* are only allowed over the slow-dynamics timescale T, since steady-state indoor-air-environment conditions are assumed.

Given *k*, let us now also introduce a local version of Equation (3):

(7) $$\begin{array}{c} \lambda_{i}=r\frac{\rho_{i}}{\Phi_{crit}},\;\;\;\;\;\rho_{i}=\frac{k_{i}}{v}, \end{array}$$

where, like $k_{i}$, $\lambda_{i}$ represents a realisation of a random variable, namely, of the exposure rate random variable, λ, which accounts for local fluctuations of the exposure rate parameter. Stated differently, λ is no longer a mere phenomenological construct (as it was considered in Section 2.2.1), but it has acquired a new, microscopic interpretation: it is distributed across *v*-sized subvolumes with some probability f(λ) that is shaped by steady-state-invariant gradients. Hence, like *k*, λ can only weakly fluctuate over $\tau_{rel}$, since large-amplitude fluctuations of λ are forbidden during T.

In light of (7), obtaining a macroscopic estimation for exposure risk statistics requires the calculation of marginal probability:

(8) $$\begin{array}{c} p\left(t\right)=\int_{0}^{\infty }d\lambda f\left(\lambda \right)p\left(t|\lambda \right), \end{array}$$

which returns the mean value of p(t|λ) over f(λ) for a given *t*, with f(λ)p(t|λ) denoting the joint probability (i.e., the probability for a pair of subsequent exposure events to be timely separated by *t* given a certain value of λ). The CDF corresponding to (8) is given by

(9) $$\begin{array}{c} P\left(t\right)=\int_{0}^{\infty }d\lambda f\left(\lambda \right)P\left(t|\lambda \right). \end{array}$$

This serves as a generalisation of (4), in the sense that it quantifies the probability for a susceptible occupant to become exposed to the virus after spending *t* hours in a heterogeneous indoor air environment.

A question that naturally arises is what an epidemiologically motivated choice for f(λ) could be. In what follows, we attempt to answer this question under the macroscopic constraint that the mean value of λ, 〈λ〉, equals $r\rho /\Phi_{crit}$, i.e., $\langle \lambda \rangle =r\rho /\Phi_{crit}$.

Plausibly, an exposure event can be thought of as the outcome of κ airborne transmissions, which do not necessarily result in the inhalation of the same amount of VCAPs. Accordingly, κ is defined as a dimensionless quantity describing the number of air samples of size *v* inhaled during $\tau^{\prime }$, i.e.,

(10) $$\begin{array}{c} \kappa :=r/\psi ,\;\;\;\;\psi =v/\tau^{\prime },\;\;\;\;\tau^{\prime }>0, \end{array}$$

where ψ (m${}^{3}$/h) re-scales *r* with respect to the epidemiologically relevant parameters *v* and $\tau^{\prime }$. Within a spatial-epidemiology context, κ is interpreted as the number of *v*-sized subvolumes surrounding a susceptible occupant, thus determining their epidemiological status (see Figure 1). Consequently, κv gives the volumetric size of an occupant’s breathing zone, i.e., $v_{br}=\kappa v=r\tau^{\prime }$. Continuing this line of thought, the κ-th surrounding subvolume is supposed to act as an airborne transmission pathway by facilitating routes through which suspended VCAPs can reach the nearest susceptible occupant. The rate at which VCAPs are transmitted via the κ-th subvolume to the nearest susceptible occupant is determined by a random variable; let it be denoted with *x* (1/h). We refer to *x* as the transmission rate, and we note that *x* essentially corresponds to the κ-th spatial component of λ, i.e., $\lambda ∼\sum_{i=1}^{\kappa }x_{i}$, $\kappa \in Z_{+}$. Introduction of *x* highlights the fact that the transmission of VCAPs is an intrinsically stochastic process that can occur over a wide range of timescales averaging 1/〈x〉, where 〈x〉>0 denotes the mean value of *x*. Assuming that the κ-th transmission event is probabilistically independent of all the others (which, in turn, implies that the κ-th pathway is not interacting with any of the other κ−1 pathways), and that the transmission of a small number of VCAPs is more likely than the transmission of a large number of VCAPs, the simplest function that can be chosen for describing the PDF of *x* across *v* is the following exponential:

(11) $$\begin{array}{c} h(x|\mu )=\mu \exp(-\mu x), \end{array}$$

where parameter $\mu =\frac{1}{\langle x\rangle }$ (h) denotes the average time separating any two transmission events and is defined as follows:

(12) $$\begin{array}{cc} \; & \mu :=\tau^{\prime }\frac{\Phi_{crit}}{\langle k\rangle }, \end{array}$$

where the ratio $\frac{\langle k\rangle }{\Phi_{crit}}$ gives the local IQ number (i.e., the average size for an IQa dose). Like λ, *x* can only weakly fluctuate over $\tau_{rel}$, so that changes in the functional shape of h(x|μ) during T are insignificant. From (12), it follows that 〈x〉 can be expressed as follows:

(13) $$\begin{array}{cc} \langle x\rangle & =\frac{\langle k\rangle }{\Phi_{crit}}\frac{1}{\tau^{\prime }}\overset{\left(7\right)}{=}\frac{K}{\Omega \Phi_{crit}}\frac{1}{\tau^{\prime }}\overset{\left(5\right)}{=}\frac{K}{V\Phi_{crit}}\frac{v}{\tau^{\prime }}\overset{\left(3\right)}{=}\frac{\rho }{\Phi_{crit}}\psi \end{array}$$

Given (10) and (13), the simplest possible functional form that one can assign to *f* so that $\lambda ∼\sum_{i=1}^{\kappa }x_{i}$ is guaranteed is that of a gamma distribution [39] of the form:

(14) $$\begin{array}{c} f\left(\lambda \right)=\frac{\mu^{\kappa }\lambda^{\kappa -1}\exp\left(-\mu \lambda \right)}{\Gamma (\kappa )} \end{array}$$

with Γ(·) being the gamma function,

(15) $$\begin{array}{c} \langle \lambda \rangle =\frac{\kappa }{\mu }=r\frac{\rho }{\Phi_{crit}} \end{array}$$

denoting the mean value of λ (note that 〈λ〉 satisfies the initially imposed macroscopic constraint), and $\mathrm{Var}\left(\lambda \right)=\frac{\kappa }{\mu^{2}}$ denoting the variance in λ. For κ=1 (14) becomes identical with (11) implying that $v_{br}=v$ (see Figure 1). When (14) is substituted into (8), we get:

(16) $$\begin{array}{c} p\left(t\right)=\frac{\kappa \mu^{\kappa }}{{\left(t+\mu \right)}^{\kappa +1}} \end{array}$$

corresponding to a Pareto Type II distribution [40] with CDF:

(17) $$\begin{array}{c} P\left(t\right)=1-{\left(1+\frac{t}{\mu }\right)}^{-\kappa }. \end{array}$$

For t=1, μ=1, and κ=1, (17) returns an exposure risk of 50% (i.e., 50% of susceptible occupants were exposed to the virus). To show that (17) serves as a generalisation of (4) one simply has to calculate P(t) while having ψ be vanishingly small, i.e.,

(18) $$\begin{array}{cc} \lim_{\psi \to 0}P\left(t\right)\; & =1-\lim_{\psi \to 0}{\left(1+\frac{t}{\mu }\right)}^{-\kappa }\\ \; & \overset{\left(10)(13\right)}{=}1-\lim_{\psi \to 0}{\left(1+\frac{\rho \psi }{\Phi_{crit}}t\right)}^{-r/\psi }\\ \; & =1-\exp(-\frac{r\rho }{\Phi_{crit}}t)\\ \; & =1-\exp(-\langle \lambda \rangle t)\equiv (4), \end{array}$$

which is achieved by taking either v→0 or $\tau^{\prime }\to \infty$.

Taking (7) and (14) together implies that *k* is realised by a rescaled (by a factor of $c=\Phi_{crit}v/r$) gamma distribution, i.e.,

(19) $$\begin{array}{c} g\left(k\right)=\frac{o^{\kappa }k^{\kappa -1}\exp\left(-ok\right)}{\Gamma (\kappa )},\;\;\;o=\frac{\mu }{c}=\frac{r\mu }{\Phi_{crit}v}\overset{\left(12\right)}{=}\frac{\kappa }{\langle k\rangle }, \end{array}$$

with the required mean value $\langle k\rangle =\frac{\kappa }{o}=\frac{K}{\Omega }$, and variance $\mathrm{Var}\left(k\right)=\frac{\kappa }{o^{2}}=\frac{{\langle k\rangle }^{2}}{\kappa }$.

### 2.3. Infection-Activation Considerations

Once inside the body, SARS-CoV-2 can enter cells located on the surface of the upper respiratory tract via binding to the angiotensin-converting enzyme 2 (ACE2) receptor [41]. High replication levels during the first hours following exposure are correlated with the risk of developing symptomatic disease [42]. Although biological details concerning infection activation remain largely unknown, knowledge gained from long-lasting superspreading events, such as the Skagit Valley Chorale choir practice [13], suggests that a prolonged exposure time increases the risk of symptomatic disease. The critical size of the cumulative viral dose that could activate an infection leading with high certainty to symptomatic disease is impossible to measure. Nevertheless, it was recently suggested that the interplay between the size of the cumulative viral dose and the efficiency of the innate immune system plays a crucial role in determining the course of infection and disease severity [43]. Anti-SARS-CoV-2 NAbs are at the frontlines of the innate immune system, since they can inhibit the binding of the virus to the ACE2 receptor, thus offering protective immunity against SARS-CoV-2 infection [36,44,45]. In this work, we assume that infection activation is a necessary but not sufficient condition for the development of symptomatic disease.

The average number of IQa inhaled after spending *t* hours in *V*, i.e., the average size for the cumulative IQa dose up to time *t*, can be estimated with

(20) $$\begin{array}{c} \Phi =\int_{t_{0}}^{t^{\prime }}dz\langle \lambda \rangle \overset{*}{=}\langle \lambda \rangle t \end{array}$$

(* is a reminder that this equality holds only under steady-state indoor-air-environment conditions). Without loss of generality, a relationship between Φ and a hypothetical NAb-orchestrated antiviral defence mechanism can be obtained via the following generalised logistic differential equation:

(21) $$\begin{array}{c} \frac{dR}{d\Phi }=aR\left(1-R^{\zeta }\right), \end{array}$$

which is known as the Richards growth model [35], where $R\in [R_{0},R_{\Phi \to \infty }=1)$ is a hypothetical infection-activation biomarker, $R_{\Phi \to \infty }$ denotes the upper asymptotic bound of *R*, $R_{0}>0$ is the initial condition for *R*, a>0 is the rate of change of *R* with respect to Φ, and ζ>0 is a dimensionless exponent accounting for host susceptibility. Concretely, ζ is considered inversely correlated with the neutralising capacity of NAbs. Hence, the larger ζ is, the smaller the neutralising capacity of NAbs is expected to be, which, in turn, implies a lower degree of protective immunity against SARS-CoV-2 infection. The solution of (21) reads

(22) $$\begin{array}{cc} \; & R={\left[1+A\exp\left(-a\zeta (\Phi -\Phi_{0})\right)\right]}^{-1/\zeta },\;\;\;A=R_{0}^{-\zeta }-1,\;\;\;\Phi_{0}=\langle \lambda \rangle t_{0}. \end{array}$$

A connection with the notion of IQ is established by requiring that the inflection point of (22)

(23) $$\begin{array}{c} \Phi_{infl}=\frac{\ln\left(\frac{A}{\zeta }\right)}{a\zeta }+\Phi_{0}, \end{array}$$

is equal to 1/ζ, i.e.,

(24) $$\begin{array}{c} \Phi_{infl}=1/\zeta \Rightarrow a=\frac{\ln\left(\frac{A}{\zeta }\right)}{1-\zeta \Phi_{0}}. \end{array}$$

$\Phi_{infl}$ designates the Φ value for which *R* attains its maximum value. Qualitatively, $\Phi_{infl}$ can be understood as the beginning (with respect to Φ) of *R*’s asymptotic convergence towards $R_{\Phi \to \infty }$. From a disease biology viewpoint, $\Phi_{infl}$ represents a threshold value of “no return” that, once surpassed, signals a high likelihood for infection activation. For ζ=1, we have that $\Phi_{infl}=1=\Phi_{crit}$, implying that one IQ suffices for infection activation. Accordingly, if ζ>1 (ζ<1), then the host is considered to exhibit low (high) NAb-attributed preparedness since $\Phi_{infl}<\Phi_{crit}$ ($\Phi_{infl}>\Phi_{crit}$). $\Phi_{infl}$ can, thus, be understood as a personalised estimation for the number of IQa required to activate an infection. This can be tuned to match an occupant’s immunological profile.

### 2.4. Indoor Infection Dynamics

Following [27], let us now claim that $\left(17\right)=\frac{E}{S}$. Then, for the initial condition $S_{0}=N-F>0$, the decrease in the number of susceptible occupants (or the increase in the number of exposed occupants) under steady-state indoor-air-environment conditions is given by

(25) $$\begin{array}{c} \frac{E}{S_{0}}=\frac{S_{0}-S}{S_{0}}=1-{\left(1+\frac{t}{\mu }\right)}^{-\kappa }\Rightarrow S=S_{0}{\left(1+\frac{t}{\mu }\right)}^{-\kappa }, \end{array}$$

which for a sufficiently small time step dt becomes the solution of the following differential equation [46,47]:

(26) $$\begin{array}{cc} \; & \frac{dS}{dt}=-\alpha S^{q}=-\frac{dE}{dt},\;\;\;\;\alpha :=\frac{\langle \lambda \rangle }{S_{0}^{q-1}},\;\;\;q:=1+1/\kappa , \end{array}$$

where α is the “effective” [46,47] exposure rate, and *q* is the Tsallis entropic index [34]. Equation (25) can be derived by maximising the Tsallis entropy functional [46,47]:

(27) $$\begin{array}{cc} \; & S\left[S\right]=-\frac{\int_{t_{0}}^{t^{\prime \prime }}S\left(1-S^{q-1}\right)dt}{1-q} \end{array}$$

subject to constraints $L_{1}=\int_{t_{0}}^{t^{\prime \prime }}S\;dt$ and $L_{2}=\int_{t_{0}}^{t^{\prime \prime }}tS^{q}\;dt$, where $L_{1}$ and $L_{2}$ are Lagrange multipliers with their values being manually adjusted so that the desired values for $S_{0}$ and 〈λ〉, respectively, may be obtained [46].

By using (22), we may now extend (26) to account for an *I* subgroup representing exposed occupants who are expected to develop symptomatic disease. We consider *R* to determine the value of a hypothetical infection rate β (1/h), delimiting the speed at which occupants are transferred from the *E* subgroup to the *I* subgroup, i.e.,

(28) $$\begin{array}{c} \beta :=\beta^{*}R, \end{array}$$

where $\beta^{*}>0$ (1/h) rescales *R* and imposes an upper asymptotic bound on β. Generally, $\beta^{*}$ can be thought of as being inversely correlated with the rate at which successful IFN-system-driven immune responses take place (for the crucial role that the IFN system can play during the first hours following infection activation, see [42]). Hence, its value determines whether an initially activated infection is sustained or not. Altogether, our modelling considerations lead us to the following set of ordinary differential equations (ODEs) describing the indoor infection dynamics of $S_{0}$ in *V*:

(29) $$\begin{array}{cc} \; & \;\;\;\;\;\;\;\;\;\;\;\;\frac{dS}{dt}=-\alpha S^{q}\\ \; & \;\;\;\;\;\;\;\;\;\;\;\;\frac{dE}{dt}=\alpha S^{q}-\beta E\\ \; & \;\;\;\;\;\;\;\;\;\;\;\;\frac{dI}{dt}=\beta E,\\ \; & \;\;\;\;\;\;\;\;\;\;\;\;\mathrm{for}\;S_{0}=N-F,E_{0}=0,I_{0}=0,\;N=\mathrm{const},\;F=\mathrm{const}. \end{array}$$

Stability analysis of the system of ODEs described in (29) is trivial; for t→∞, all occupants were expected to have been transferred to the *I* subgroup, i.e., $\left(S_{t\to \infty }\to 0,\;E_{t\to \infty }\to 0,\;I_{t\to \infty }\to N-F\right)$ is the unique equilibrium point globally attracting from within the positively invariant region {(S,E,I)|S+E+I≤N−F}.

The presented SEI model belongs, from a mathematical point of view, to the class of *q*-SEIR models recently introduced in [19].

The numerical integration of (29) was performed in Python [48] by employing a Runge–Kutta method of order 5(4) [49].

## 3. Insights Gained from Computational Analysis

### 3.1. Scrutinising the Generalised WRIP

To better understand the implications for indoor biosafety stemming from (17), we may focus on (18). We set r=1, and considered the case where *K* VCAPs were suspended in *V* with 〈λ〉=1 so that $\frac{Fw}{W\Phi_{crit}}=\frac{\rho }{\Phi_{crit}}=1$. With this choice of parameters, we gain some insight into how (17) approaches (4), and how f(λ) and g(k) behave for decreasing the transmission range ξ while keeping the exposure time $\tau^{\prime }$ (and, thus, also $v_{br}$) fixed.

**Figure 2:**
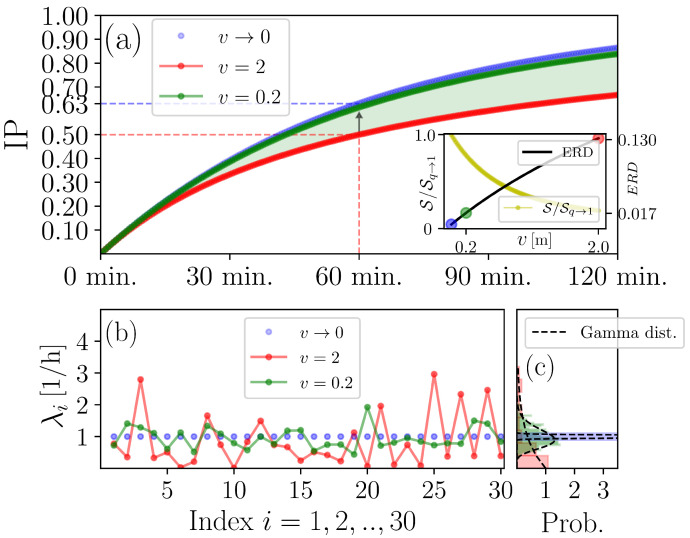
Classical WRIP as an upper asymptotic bound of its generalisation. (**a**), we plot (17) for μ=1/ψ and κ=1/ψ with $\tau^{\prime }=\tau =2$ and v∈(0,2] (i.e., ψ∈(0,1]). The black arrow indicates that the generalised WRIP measure approaches the classical one as *v* decreases. The shaded area between the two curves visualises the image of the generalised WRIP function for ψ∈(0,1]. An estimation of the exposure risk difference (ERD), i.e., the difference between the generalised and the classical WRIP measures, calculated by ${\left(1+\frac{t}{\mu }\right)}^{-\kappa }-\exp\left(-t\right)$ for v∈(0,2] and t=1 (=60 min) is shown in the inset graph. Colorful markers in the inset graph indicate the *v* key values considered in (**a**). In the same inset graph, the ratio $S/S_{q\to 1}$ is plotted, where S is calculated by using the discretised version of (27) and with $S_{q\to 1}$ denoting the Boltzmann–Gibbs entropy (see Appendix A.3, Equations (A3) and (A4), respectively). (**b**), for a hypothetical volume of size V=60, we plot i=1,2,…,30 randomly-chosen realisations of λ for v→0, v=0.2, and v=2 implying that Ω→∞, Ω=300, and Ω=30, respectively, since $v=\frac{V}{\Omega }$ (see (5)). (**c**), The corresponding gamma distributions are shown. Different colors are used to visualise the distributions obtained for the key values appearing in (**a**,**b**).

As we can see in Figure 2a, for a vanishingly small ξ (i.e., v→0), (4) approaches its upper asymptotic bound, which is given by (17). Simply put, the classical WRIP is an overestimation of the exposure risk justified on the basis that, in the absence of any substantial knowledge concerning fluctuations of λ, the worst-case scenario may be assumed, namely, that any realisation of λ would be approximately equal to 〈λ〉. The necessary condition supporting this simplification is that the VCAP density is very large, i.e., *K* and *V* should be very large and small, respectively (see also Appendix A.1). On the other hand, the generalised WRIP returns a lower but more realistic estimation for the exposure risk on the basis of the expectation that f(λ) may be well-approximated in terms of a gamma distribution. In fact, (17) serves as a more “fair” IIRE, in the sense that the chance for an occupant to become exposed to the virus after spending one hour in *V* is the same as tossing an unbiased coin, namely, 50% (see Figure 2a). In particular, the difference between the classical and the generalised WRIP at t=1 decreases from ≈0.13 to ≈0.01 if one considers a tenfold increase in the number of subvolumes Ω (see Figure 2b and the corresponding legend text).

**Figure A1:**
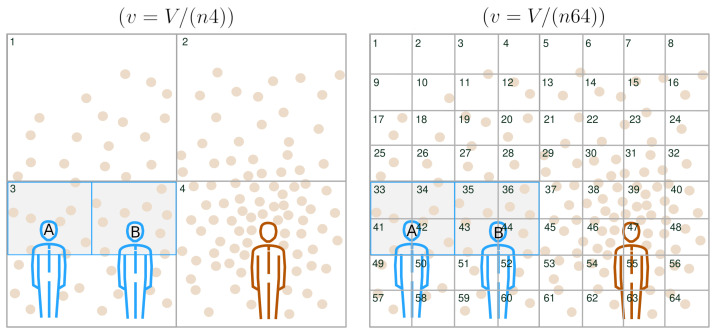
Investigating contaminated-air-sharing scenarios. We consider the same setup as that in Figure 1, but for *V* partitioned in two different ways. For simplicity, we assume that $v=\xi^{3}$. The susceptible occupants’ breathing zones are highlighted and outlined in light grey and blue, respectively. On the **left** subfigure, we partitioned *V* into i=1,2,…,Ω=n4 subvolumes of size v=V/Ω, so that $v_{br}=v/4$, implying that $\xi >\sqrt[{3}]{v_{br}}$. The susceptible occupants’ breathing zones were embedded within the subvolume indexed with i=3. Because $\xi >\sqrt[{3}]{v_{br}}$, the epidemiological status of *A* and *B* may be influenced by any of the surrounding subvolumes i=1,2,3,4. In fact, during $\tau_{rel}$, a VCAP suspended within any of i=1,2,3,4 could reach *A* or *B*. Hence, it is likely that *A* and *B* are sharing contaminated air; imagine a VCAP originating from any of i=1,2,3,4 being displaced towards and inhaled by either *A* or *B* during $\tau_{rel}$. On the **right** subfigure, we partitioned *V* into i=1,2,…,Ω=n64 subvolumes of size v=V/Ω, so that $v_{br}=4v$, implying that $\xi <\sqrt[{3}]{v_{br}}$. Those indexed with i=33,34,41,42 and i=35,36,43,44 were embedded within the breathing zone of *A* and *B*, respectively. Because $\xi <\sqrt[{3}]{v_{br}}$, the epidemiological status of *A* and *B* is unlikely to be influenced by any other surrounding subvolumes except from i=33,34,41,42 and i=35,36,43,44, respectively. Hence, *A* and *B* are also unlikely to share contaminated air, since VCAPs suspended in any of i=33,34,41,42 and i=35,36,43,44 are unlikely to reach *B* and *A*, respectively, during $\tau_{rel}$.

The information content of an *S* trace is measured in terms of *q* entropy S (see Equation (27)) that, as we can see in the inset of Figure 2a, is inversely correlated with ξ. The loss of *S*-trace-related information with increasing ξ accounts for the degree of viral dispersity in *V* quantified in terms of the reciprocal of κ, $1/\kappa \propto \frac{\xi^{3}}{v_{br}}$. Intuitively, we may understand 1/κ as a rough indicator for the likelihood that a suspended VCAP misses its target due to either the smallness of $v_{br}$ or the largeness of ξ (or both). Of particular interest is the case where ξ is large since, as we show in Figure A1 found in Appendix A.2, it may support a contaminated-air-sharing scenario where a pair of susceptible occupants are competing for the same IQa dose, i.e., a suspended VCAP could potentially reach any two susceptible occupants during $\tau_{rel}$. Thus, one might anticipate that *S*-trace-related information losses associated with a composite epidemiological system A⊕B (where *A* and *B* may represent any two susceptible occupant subgroups (or “subsystems”)) should be proportional to 1/κ. Indeed, because S is nonadditive, we have that

(30) $$\begin{array}{cc} S[A⊕B] & =S[A]+S[B]-(1-q)S[A]S[B]\\ \; & =S\left[A\right]+S\left[B\right]-\frac{S[A]S[B]}{\kappa }\Rightarrow \\ S[A⊕B] & -\left(S\left[A\right]+S\left[B\right]\right)\propto \frac{\xi^{3}}{v_{br}}S\left[A\right]S\left[B\right], \end{array}$$

where $A:=\{p_{i}^{\left(A\right)}\}$, $i=1,2,\ldots ,W_{A}$, $B:=\{p_{j}^{\left(B\right)}\}$, $j=1,2,\ldots ,W_{B}$, and $A⊕B:=\{p_{i,j}^{\left(A⊕B\right)}=p_{i}^{\left(A\right)}p_{j}^{\left(B\right)}\}$ are time-step-specific probabilities of escaping exposure introduced while discretising S (see Appendix A.3, Equation (A3)), and the term $\frac{S[A]S[B]}{\kappa }$ quantifies the losses in *S*-trace-related information due to potential realisation of a contaminated-air-sharing scenario involving *A* and *B* as a pair. This is summarised in terms of the Tsallis entropic index *q*: for v→0 (i.e., 1/κ→0), we have that q→1, indicating that indoor-air-environment homogeneity is restored. In turn, this implies that the information content of *S* is maximised, i.e., the Boltzmann–Gibbs entropic functional is recovered (see Appendix A.3, Equation (A4), and Figure 2b), and the likelihood of pairwisely sharing contaminated air is negligible.

To gain more insight into how steady-state-invariant gradients shape the statistics of λ and *k*, the asymptotes of f(λ) and g(k), respectively, are deduced. First, for v≪1, we have that

(31) $$\begin{array}{cc} \; & \sqrt{\mathrm{Var}(\lambda )}\ll \langle \lambda \rangle \;\;\;\;\left(a\right)\\ \; & \sqrt{\mathrm{Var}(k)}\ll \langle k\rangle \;\;\;\;\left(b\right) \end{array}$$

since 〈λ〉∝v (because 〈λ〉 is macroscopically constrained by construction) and $\sqrt{\mathrm{Var}(\lambda )}\propto v$, and 〈k〉∝v and $\sqrt{\mathrm{Var}(k)}\propto v^{3}$ apply, respectively. Inequality (a) in (31) implies that f(λ) resembles a Gaussian distribution of the form [50]:

(32) $$\begin{array}{c} \frac{\mu^{\kappa }}{\Gamma (\kappa )}\exp\left(-\frac{\mu^{2}}{2\kappa }{\left(\lambda -\langle \lambda \rangle \right)}^{2}\right)\propto \frac{v^{-1/v}}{\Gamma (1/v)}\exp\left(-\frac{1}{2v^{2}}{\left(\lambda -\langle \lambda \rangle \right)}^{2}\right) \end{array}$$

(see Figure 2c, green distribution), and, eventually, approaches the Dirac delta distribution located at 〈λ〉, i.e.,

(33) $$\begin{array}{c} f_{v\to 0}\left(\lambda \right)=\delta \left(\lambda -\langle \lambda \rangle \right) \end{array}$$

is the asymptote of f(λ) (see Figure 2c, blue distribution). Equation (33) sets the basis for constructing classical WRIPs (see Figure 2c). In fact, plugging $f_{v\to 0}\left(\lambda \right)$ into (9) gives (4).

**Figure 3:**
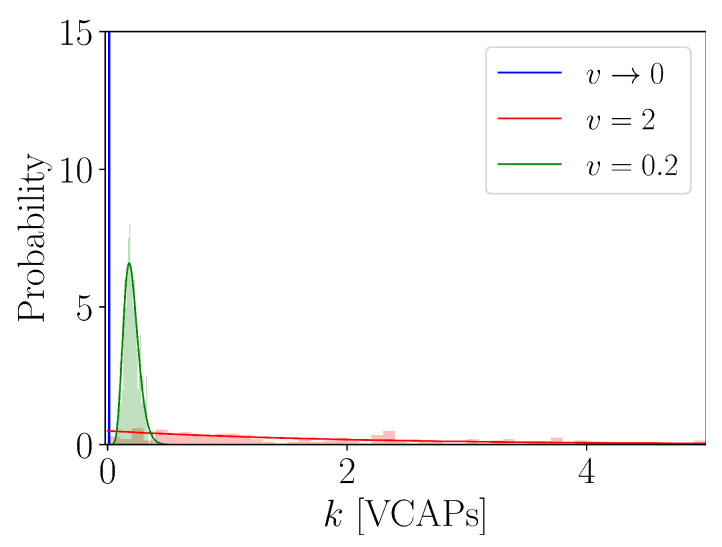
Instances of a VCAP distribution. We plot g(k) for μ=1/ψ and κ=1/ψ with $\tau^{\prime }=\tau =2$ and v∈(0,2] (i.e., ψ∈(0,1]).

Let us now turn our attention to Inequality (b) in (31). Its main implication is the same as previously: g(k) can be approximated with the Gaussian distribution of the form [50]:

(34) $$\begin{array}{c} \frac{o^{\kappa }}{\Gamma (\kappa )}\exp\left(-\frac{o^{2}}{2\kappa }{\left(k-\langle k\rangle \right)}^{2}\right)\propto \frac{v^{-2/v}}{\Gamma (1/v)}\exp\left(-\frac{1}{2v^{3}}{\left(k-\rho v\right)}^{2}\right) \end{array}$$

(see Figure 3, green distribution), and, eventually, approaches the Dirac delta distribution located at zero, i.e.,

(35) $$\begin{array}{c} g_{v\to 0}\left(k\right)=\delta \left(k\right) \end{array}$$

is the asymptote of g(k) (see Figure 3, blue distribution). Equation (35) tells us that *V* is partitioned into an infinite number of subvolumes, each containing an infinitesimally small number of VCAPs. This is because the mean value and variance of g(k) are proportional to *v* and $v^{3}$, respectively, so that g(k) is translocated towards the origin while at the same time becoming increasingly narrow as *v* decreases for some finite value of *K* (see Figure 3). For v→0, it is, thus, almost certain to find an infinitesimally small amount of VCAPs anywhere in *V* as if VCAPs were molecules of a well-mixed gas [27].

### 3.2. Refining the IIRE

**Figure 4:**
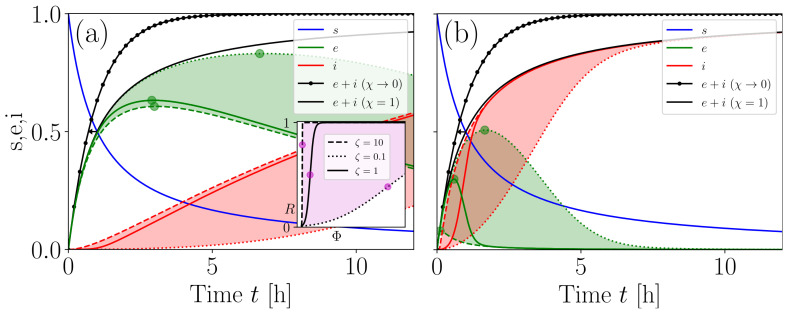
Refined biosafety scoring. (**a**), *s*, *e*, *i* traces are plotted for μ=1/ψ and κ=1/ψ with $v=\tau^{\prime }=\tau =12$ (i.e., ψ=1), $\beta^{*}=0.1$, $R_{0}=0.01$, and ζ∈[0.1,10]. The shaded areas highlight the images of the *E* and *I* for ζ∈[0.1,10]. Green circular markers highlight the maxima of *E* (their values and locations both decreased with increasing β, indicating that the transfer of occupants from the *E* subgroup to the *I* subgroup was accelerated). The black arrow indicates that the sum E+I approached the classical WRIP for v→0. Φ-dependent dynamics of *R* are illustrated in the inset graph. Magenta points in the inset graph highlight *R* values corresponding to the tunable inflection point $\Phi_{infl}=1/\zeta$. The shaded area in the inset graph visualises the image of *R* for ζ∈[0.1,10]. The main and inset graphs use the same line styles to account for ζ=0.1,1,10. (**b**), same as (**a**), but for $\beta^{*}=10$.

Of particular epidemiological interest is understanding how IIREs depend not only on indoor-air-environment properties, but also on personalised immunological traits. Towards this end, we may utilise the SEI model described by (29) as a simple tool to probe different scenarios. In Figure 4, we demonstrate how our model refines the IIRE procedure in the sense that the classical WRIP is now represented in terms of two additive components incorporating out-of-host and within-host information, namely, *E* and *I*, respectively, so that $E+I\to S_{0}\left(1-\exp\left(-\langle \lambda \rangle t\right)\right)$ for v→0. Normalising SEI traces over $S_{0}$ turns them into personalised biosafety scores returning the *t*-dependent probabilities $\left\{s:=S/S_{0},e:=E/S_{0},i:=I/S_{0}\right\}$ of escaping exposure, being exposed, and getting infected (i.e., developing symptomatic disease sometime in the near future), respectively. Decreasing the value of the host susceptibility parameter ζ translocates the *I* trace towards the origin, thus decelerating the transfer of occupants from the *E*-subgroup to the *I*-subgroup (see Figure 4 and the corresponding legend text) since infection activation is efficiently suppressed due to a high degree of protective immunity. This reflects the action of NAbs and is manifested as a decelerated increase in a hypothetical infection-activation biomarker, *R* (see (22)), induced by translocating inflection point $\Phi_{infl}$ away from the origin as ζ decreases (see the inset graph in Figure 4a). As one might expect, the rate of IFN-system-driven successful responses, $\beta^{*}$, sets the speed at which occupants are transferred from the *E* subgroup to the *I* subgroup (compare Figure 4a and Figure 4b). In principle, however, the value of $\beta^{*}$ is unknown in the IIRE procedure; it may exhibit nontrivial time dependencies over viral replication dynamics and the host’s immunological profile.

### 3.3. Evaluation of the Six-Foot Rule

We now demonstrate how our modelling procedures can aid in designing and testing distance-based mitigation strategies specifically targeting indoor spaces. We focused on how changes in the $\left\{\kappa ,\zeta ,\beta^{*}\right\}$ triplet affect SEI dynamics under different steady-state VCAP density conditions. As a concrete example, we scrutinise the effectiveness of the six-foot rule, which dictates that the maximum number of occupants should be $N_{max}=\sqrt{length\cdot width}/d_{safe}$, V=length·width·height, where $d_{safe}=1.8$ (≈6 ft) is the minimal distance to be kept at all times between any two occupants to guarantee biosafety. To cover the worst-case scenario, we require that (a) the average maximum distance over which virions can be transmitted is determined by the minimal distance separating any pair of occupants, i.e., $\xi =d_{safe}$, and (b) the breathing zones of susceptible occupants are uninterruptedly contaminated, i.e., we set $\tau^{\prime }=\tau$, so that ψ was minimised. On this basis, we proceeded with collecting $i_{\tau }:=i\left(t=t^{\prime \prime }\right)$ values for τ=0.25,0.5,0.75,1, and r=0.54,1.38,3.30 (breathing rates values correspond to the mean values reported for the activities of standing, light exercise, and heavy exercise, respectively [51]), and analyse their functional dependence over ρ for different values of $\left\{\zeta ,\beta^{*}\right\}$.

**Figure 5:**
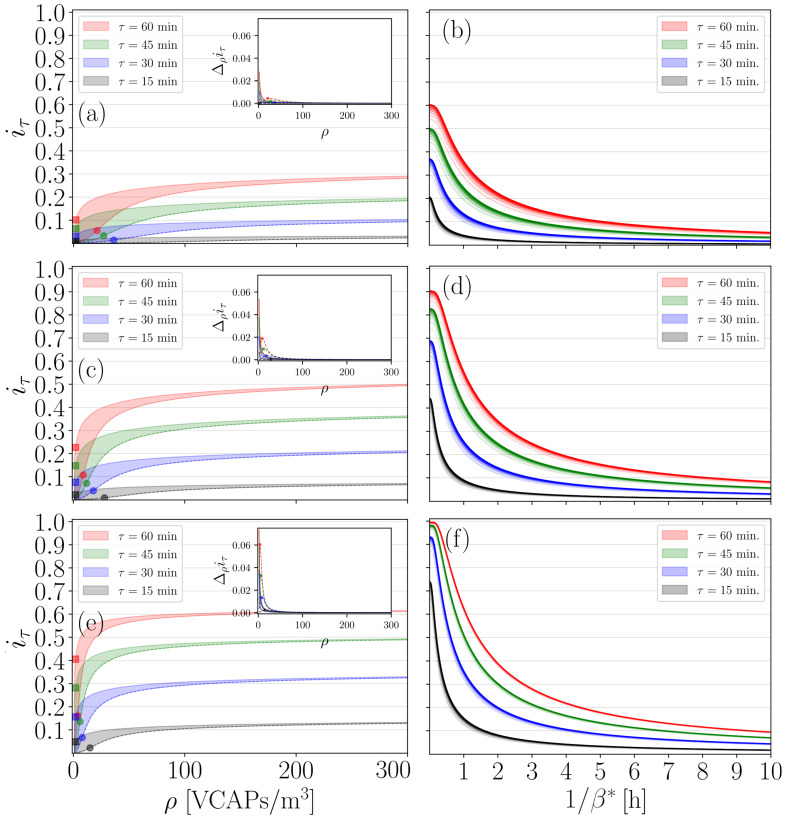
Evaluation of the six-foot rule. (**a**), we plot $i_{\tau }$ as a function of ρ for r=0.54, $\zeta \in [\zeta_{min}=0.1,\zeta_{max}=10]$, $\beta^{*}=1$, $\xi =d_{safe}$, $R_{0}=0.01$, and τ=0.25,0.5,0.75,1. Continuous and dashed lines highlight the upper and lower boundaries of $i_{\tau }$ values range obtained for $\zeta_{min}=0.1$ and $\zeta_{max}=10$, respectively. (**c**), same as (**a**), but for r=1.38. (**e**), same as (**a**), but for r=3.3. Inset graphs in (**a**,**c**,**e**) illustrate how the first-order differences trace $\Delta_{\rho }i_{\tau }=i_{\tau ;\rho +\Delta \rho }-i_{\tau ;\rho }$ “flattens” for increasing ρ. The following computational criterion was used to decide whether a $\Delta_{\rho }i_{\tau }$-trace has “flattened”: find $\rho^{*}$ so that $\Delta_{\rho }i_{\tau }<\epsilon$, $\epsilon =1\times 10^{-4}$, is satisfied for any $\rho \geq \rho^{*}$. Once all $\rho^{*}$ values had been gathered for a specific *r*-value, we found their maximum, $\rho_{max}^{*}=\max_{\zeta ,\tau ,\beta^{*}=1}\left\{\rho^{*}\right\}$. $\rho_{max}^{*}$ is provided here for convenience: it serves as a baseline value for probing the behaviour of $i_{\tau }$-values with respect to $1/\beta^{*}$. Round and square markers in (**a**,**c**,**e**) highlight the values of $i_{\tau }$ at the inflection point $\rho_{infl}$ for $\zeta =\zeta_{min}$ and $\zeta =\zeta_{max}$, respectively. For clarity, only round markers are used in the inset graphs, highlighting the maxima of $\Delta_{\rho }i_{\tau }$ for $\zeta =\zeta_{min}$. (**b**) We plot the value of $i_{\tau }$ obtained for $\rho =n\rho_{max}^{*}$, n=1,2,3,…,10, $\rho_{max}^{*}=384$ versus $1/\beta^{*}$ for r=0.54, $\zeta \in [\zeta_{min},\zeta_{max}]$, and τ=0.25,0.5,0.75,1. (**d**), same as (**b**), but for r=1.38 with $\rho_{max}^{*}=354$. (**f**), same as (**b**), but for r=3.3 with $\rho_{max}^{*}=245$. Different colour intensities in (**b**,**d**,**f**) were used to visualize $i_{\tau }$-values in ascending *n*-order. Black traces appearing in (**b**) are particularly interesting here, as they indicate that for exposure times as short as 15 min, $i_{\tau }$ can take values as high as ≈0.2 if the host’s innate immune system falls short in counteracting the viral threat, i.e., if $1/\beta^{*}$ is very small.

First, we focus on how $i_{\tau }$ traces behave as functions of ρ for $\zeta \in [\zeta_{min},\zeta_{max}]$ and $\beta^{*}=1$. We rely on the existence of an inflection point $\rho_{infl}$ marking the location where the change from convex to concave in an $i_{\tau }$ trace takes place for increasing ρ. For small values of ζ (strong protective immunity), $\rho_{infl}$ is translocated away from the origin, thus decelerating the increase in $i_{\tau }$ (see Figure 5a,c,e). On the other hand, for large values of ζ (weak protective immunity), $i_{\tau }$ exhibits a steep increase for $\rho <\rho_{infl}$ with $\rho_{infl}$ being located very close to the origin (see Figure 5a,c,e). For $\rho >\rho_{infl}$, a saturation $i_{\tau }$ plateau is gradually formed due to epidemiological spatiotemporal constraints imposed by the magnitude of ψ (see Figure 5a,c,e and the corresponding inset graphs). Specifically, there is a large value of ρ, let this be $\rho^{*}>\rho_{infl}$, for which $i_{\tau }$ traces converge (i.e., the range within which $i_{\tau ;\rho \geq \rho^{*}}$ values lie tends to be vanishingly small) irrespective of what the value of ζ is (see Figure 5a,c,e, and the corresponding inset graphs). This regime emerges when local VCAP densities are so high that protective immunity is inadequate in counteracting the viral threat, i.e., $k_{i}\gg \Phi_{infl}$; consequently, the influence of ζ on the SEI dynamics is rendered marginal. In computational practice, $\rho^{*}$ is obtained by finding the value of ρ for which the first-order differences of $i_{\tau }$ drop below a threshold (for details, see the legend of Figure 5).

Lastly, we evaluate the performance of the six-foot rule by ensuring again that our calculations covered the worst-case scenario. Namely, we required that $\rho \geq \rho^{*}$ and $\beta^{*}\;\to \;\infty$, implying that both NAb-orchestrated and IFN-system-driven lines of defence cannot provide sufficient protection against a viral threat. Figure 5b shows that, if $N_{max}$ occupants spend 15, 30, 45, and 60 min in *V* standing, then $i_{\tau }$ could attain values as high as ≈0.2, ≈0.35, ≈0.5, and ≈0.6, respectively. Crucially, this finding shows that the efficiency of the six-foot rule drops significantly after 15 min, since susceptible occupants with a weak immune system might face as high a symptomatic-disease risk as ≈20% (notice the behaviour of the black traces shown in Figure 5b for very small $1/\beta^{*}$). If $N_{max}$ occupants spend time in *V* while engaging in light exercise activities, then $i_{\tau }$ could potentially exceed 0.4, 0.6, 0.8, and 0.9, respectively (see Figure 5d). Moreover, if $N_{max}$ occupants engage in heavy exercise activities, then $i_{\tau }$ can attain values larger than 0.7 and 0.9 for τ=0.25 and τ=0.5,0.75,1, respectively, as we can see in Figure 5f. The last two cases suggest that the six-foot rule cannot guarantee the biosafety of immunologically weak susceptible occupants in indoor spaces where exercise activities are undertaken, even if the exposure time is shorter than 30 min. Obviously, for $\beta^{*}\;\to \;0$, we have that $i_{\tau }\;\to \;0$, since the transfer from the *E* to the *I* subgroup is suppressed as if the IFN-system-driven response would successfully disrupt viral replication and eliminate the virus (see Figure 5b,d,f).

## 4. Discussion

Given the current rate of biosphere degradation, respiratory viruses of zoonotic origin capable of spreading via air, such as SARS-CoV-2, are expected not only to emerge more often, but also to carry an unprecedentedly high epidemic potential. Self-evidently, the probability of infection increases significantly indoors due to the very nature of the built environment, that is, enclosure. Thus, the biosafety of societies whose members spend most of their time indoors is likely compromised.

In this work, we presented a minimal (in terms of parameter space size) ODE-based model for describing indoor exposure to and potentially also infection by an airborne-transmitted virus. The initial motivation for this work was to construct a modelling framework that could stretch beyond the idea of environmental and biological homogeneity within the context of indoor air biosafety. We achieved this by generalising the WRIP on the basis of a κ-pathway spatial model that treats the breathing zone as a stochastic VCAP transmitter. Following this line of thought, we deduced that the most general PDF that could be used to statistically describe the spatial fluctuations of the exposure rate parameter and VCAP number is that of a gamma distribution. This pointed towards a Tsallisian entropic origin of transmission dynamics, with *q* measuring the departure from the homogeneous case. The connection between Tsallis entropy and superstatistics is usually established via $\chi^{2}$ distribution (e.g., see [33]) which is nothing but a special case of the gamma distribution. Lastly, by extending exposure dynamics to account for the possibility of infection activation in relation to innate-immune-system defence mechanisms, we propose that *i* (defined in Section 3.2) can be used as a probability measure for estimating the risk for developing symptomatic COVID-19 disease. Although Tsallis entropy-based modelling approaches have proven useful in understanding the spread of SARS-CoV-2 among the general population (e.g., see [19,52]), the model curated in this work serves as a first attempt to zoom in on a single indoor space. We emphasise that even for a 15 min exposure under the six-foot-rule guideline, a symptomatic-disease risk as high as 20% might apply.

Let us now discuss some of the limitations of this work. First, theoretical predictions concerning the VCAP distribution remain to be verified (or dismissed) by CFD simulations. In practice, the values of parameters {κ,μ} can be estimated by analysing steady-state VCAP distributions obtained from CFD experiments by employing standard distribution parameter estimation procedures. If {r,w,W} are known, then one can investigate which combinations of *v* and $\tau^{\prime }$ offer the best description for the experimental PDF of *k*. Inevitably, rigorous definitions for $\tau^{\prime }$ and ξ then have to be provided in accordance with the simulation details. Second, very little is known about how the innate immune system reacts to SARS-CoV-2. Therefore, the presented extension of exposure dynamics based on parameter pair $\left\{\zeta ,\beta^{*}\right\}$ serves as a rather general starting point for investigating the role of immunologically heterogeneous responses to SARS-CoV-2 and might be updated in the future as our knowledge increases. In particular, future studies could explore the possibility of $\beta^{*}:=\beta^{*}\left(t\right)$ in order to probe the relationship among IFN-system-driven immune responses and SARS-CoV-2 replication dynamics.

Overall, our work demonstrates how superstatistics and related *q*-entropies can open up possibilities for systematically obtaining exposure and symptomatic-disease risk estimations in heterogeneous indoor air environments. In turn, this allows for minimising the number of parameters used when constructing personalised biosafety scores such as *i*. Therefore, our work provides a recipe for incorporating VCAP-related spatial information into simple ODE-based models for indoor epidemiological scenario explorations.

## 5. Conclusions

At the macroscopic level, the classical Wells–Riley infection probability results in an overrated exposure risk estimation. Locally, however, the situation is different: a classical Wells–Riley infection probability can either over- or under-estimate the associated risks depending on whether the corresponding realisation of exposure rate parameter $\lambda_{i}$ is larger or smaller, respectively, than its mean value 〈λ〉.

The Tsallis entropic functional can serve as an information-theoretical starting point for exploring SEI dynamics in heterogeneous indoor air environments.

## Figures and Tables

**Table 1:** We consider two parameter sets, namely, the out-of-host set $\left\{N,F,V,v_{br},\tau ,r,w,W,\xi ,\tau^{\prime }\right\}$ and the within-host set $\left\{\Phi_{crit},\zeta ,\beta^{*}\right\}$. Out-of-host parameters *r*, *w*, and *W* denote the volumetric inhalation rate, the VCAP exhalation rate, and the ventilation rate, respectively, and are introduced in Section 2.2.1. Within-host parameters ζ and $\beta^{*}$ codetermine a susceptible occupant’s immunological profile and are formally introduced in Section 2.3. λ, ρ, κ, and μ may be considered summary parameters, as they are expressed as combinations of out-of- and within-host parameters. λ and ρ represent the exposure rate parameter and VCAP density, respectively, and are introduced and reinterpreted in Section 2.2.1 and Section 2.2.2, respectively. κ denotes the number of local air environments (modelled as surrounding subvolumes of volumetric size $\propto \xi^{3}$) determining a susceptible occupant’s epidemiological status and is introduced in Section 2.2.2. μ gives the average time separating any pair of subsequent airborne transmission events and is introduced in Section 2.2.2.

Summary Param.	Out-of-Host Param.	Within-Host Param.
$\lambda =\frac{rwF}{W\Phi_{crit}}$ (1/h)	*N* (nr. of occupants)	$\Phi_{crit}$ (VCAPs)
$\rho =\frac{Fw}{W}$ (VCAPs/m${}^{3}$)	*F* (nr. of infectors)	ζ (dimensionless)
$\kappa \propto \frac{v_{br}}{\xi^{3}}$ (dimensionless)	*V* (m${}^{3}$)	$\beta^{*}$ (1/h)
$\mu \propto \frac{\Phi_{crit}}{\rho }\frac{\tau^{\prime }}{\xi^{3}}$ (h)	$v_{br}$ (m${}^{3}$)	
	τ (h)	
	*r* (m${}^{3}$/h)	
	*w* (VCAPs/h)	
	*W* (m${}^{3}$/h)	
	ξ (m)	
	$\tau^{\prime }$ (h)	

## Data Availability

Not applicable.

## Appendix A

### Appendix A.1. Probing the Spatial Configuration of VCAPs

To probe the geometry underlying a steady-state VCAP distribution, one may adopt a moments-expansion scheme borrowed from molecular field theory [53]. In fact, moments-expansion schemes serve as simple, yet, informative tools for studying the spatial organisation of heterogeneous molecular systems [54,55]. Imposing periodic boundary conditions over *V*, we utilise the following geometrical nonuniformity index:

(A1) $$\begin{array}{cc} \; & \gamma :={\langle \frac{1}{K}\sum_{i}^{K}∥h_{i}\left(t\right)∥\rangle }_{t^{\prime }}\;\;\;\;\left(m\right),\\ \; & h_{i}\left(t\right)=\frac{1}{K^{\prime }}\sum_{j}^{K^{\prime }}(\overset{=d_{ij}\left(t\right)\;\;\left(m\right)}{\overset{︷}{p_{j}\left(t\right)-p_{i}\left(t\right)}})\;\;\;\;\left(m\right), \end{array}$$

where $p_{i}$ is a *t*-dependent vector from the coordinate system origin to the location of the *i*-th VCAP, $d_{i,j}$ is a *t*-dependent vector difference between the *i*-th and *j*-th VCAP locations, $h_{i}$ is the first-order geometric moment expanded about the *i*-th VCAP location calculated and normalised over $0<K^{\prime }\leq K-1$ nearest-neighboring VCAP locations, ∥·∥ is the Euclidean norm, and ${\langle \;\cdot \;\rangle }_{t^{\prime }}$ returns the mean value over all $t^{\prime }$ instances (i.e., ensemble average). $h_{i}$ serves here as a *t*-dependent measure of the displacement tendency (or “imbalance” [53]) of the *i*-th VCAP about its current location, capturing the magnitude and direction of local VCAP density fluctuations. If VCAPs tend to be uniformly spaced in *V* (which is a plausible scenario if *K* and *V* are very large and small, respectively), then we expect that $\left|\right|h_{i}\left|\right|\approx 0$ for $K^{\prime }$ would exceed a lower-value threshold value. In turn, this implies that γ→0, thus making the case for a homogeneous indoor air environment. On the other hand, for heterogeneous indoor air environments, γ is larger than zero, letting its maximum value delimited by the diagonal of *V* be *D*, i.e., γ∈[0,D). Given γ, nothing can really be said about ξ except that

(A2) ξ∝γ.

Proportional relationship (A2) implies that a nonvanishing value for γ sets the ground for long-range airborne transmission since the displacement of VCAPs is possible. On the other hand, for γ→0, the range of airborne transmission is expected to be predominantly short; large-magnitude displacements of VCAPs in homogeneous indoor air environments are unlikely to take place.

### Appendix A.3. Discretisation of the Tsallis Entropic Functional

The following discretised version of (27) was used:

(A3) $$\begin{array}{cc} \; & S\left[\left\{p_{i}\right\}\right]=-\frac{1-\sum_{i}p_{i}^{q}}{1-q},\;\;\;p_{i}=\frac{s_{i}}{\sum_{i}s_{i}} \end{array}$$

with $s_{i}:=S\left(t_{i}\right)$, $t_{i=1,2,\ldots \;,W}=t_{0}+\frac{i-1}{W-1}\left(t^{\prime \prime }-t_{0}\right)$, where $p_{i}\propto {\left(1+\frac{t}{\mu }\right)}^{-\kappa }\overset{\left(17\right)}{=}1-P\left(t\right)$ is a time-step-specific probability of escaping exposure (i.e., not inhaling any VCAPs) after spending *t* hours in *V*. $\sum_{i=1}^{W}p_{i}=1$ with *W* being identified as the number of accessible microstates. Accordingly, a subgroup of susceptible occupants of size smaller than $S_{0}$ may be thought of as a subsystem visiting the *i*-th microstate with some probability $p_{i}$. The probabilistic independence of any pair of subsystems is guaranteed by model construction, since probabilistically independent airborne transmissions are assumed throughout. For q→1, (A3) gives us the Boltzmann–Gibbs entropy, i.e.,

(A4) $$\begin{array}{c} S_{q\to 1}\left[\left\{p_{i}\right\}\right]=-\sum_{i=1}^{W}p_{i}\ln\left(p_{i}\right). \end{array}$$
